# Exosomal MicroRNA let-7 Modulates Lipid Metabolism and Inflammation in Foamy Macrophages of Chronic Obstructive Pulmonary Disease

**DOI:** 10.3390/ijms26188800

**Published:** 2025-09-10

**Authors:** Miao-Hsi Hsieh, Ping-Fang Lai, Pei-Chi Chen, Xiao-Ling Liu, Wei-Leng Chen, Wen-Shuo Kuo, Shulhn-Der Wang, Hui-Fang Kao, Li-Jen Lin, Lawrence Shih-Hsin Wu, Jiu-Yao Wang

**Affiliations:** 1 Research Center of Allergy, Immunology and Microbiome (A.I.M.), China Medical University Hospital, China Medical University, Taichung 404327, Taiwan; karinadrift@gmail.com (M.-H.H.); chenn9143@gs.ncku.edu.tw (P.-C.C.); activesitess@gmail.com (W.-S.K.); 2 Institute of Biochemistry and Molecular Biology, College of Medicine, National Cheng Kung University, Tainan 701401, Taiwan; vint822@gmail.com; 3 Department of Microbiology & Immunology, College of Medicine, National Cheng Kung University, Tainan 701401, Taiwan; 4 School of Chemistry and Materials Science, Nanjing University of Information Science and Technology, Nanjing 210044, China; 5 School of Post-Baccalaureate Chinese Medicine, China Medical University, Taichung 40402, Taiwan; 6 Department of Nursing, National Tainan Junior College of Nursing, Tainan 700007, Taiwan; 7 School of Chinese Medicine, China Medical University, Taichung 40402, Taiwan; linlijen@mail.cmu.edu.tw; 8 Institute of Biomedical Sciences, China Medical University, Taichung 40402, Taiwan; 9 Department of Allergy and Immunology, China Medical University Children’s Hospital, China Medical University, Taichung 404327, Taiwan

**Keywords:** COPD, foamy macrophages, exosomes, microRNA, let-7, lipid metabolism, inflammation, PPAR/RXR

## Abstract

Chronic obstructive pulmonary disease (COPD) involves persistent inflammation and dysregulated lipid metabolism, with foamy macrophages playing a central role in disease progression. Exosomes—vesicles transporting microRNAs (miRNAs)—mediate intercellular communication, but their contribution to foamy macrophage-driven COPD remains unclear. This study investigates the role of exosomal miRNAs, particularly let-7, in modulating lipid metabolism and inflammation in foamy macrophages. Bone marrow-derived macrophages (BMDMs) were treated with oxidized low-density lipoprotein (oxLDL) and lipopolysaccharide (LPS) to induce foamy macrophage formation. Exosomal miRNA profiles were analyzed, and the function of let-7c-3p was assessed via transfection. Foamy macrophages released significantly more exosomes (392.7 × 10^7^ particles) than controls (284.9–302.5 × 10^7^), without differences in exosome size or molecular content. The miRNA sequencing and qRT-PCR confirmed downregulation of exosomal let-7c-3p in foamy macrophages, correlating with increased RNF8 and decreased RXR expression—markers of disrupted PPAR/RXR signaling. Pathway analysis implicated let-7c-3p in regulating PPAR/RXR, WNT/β-catenin, and pulmonary fibrosis pathways. Transfection with let-7 mimics reduced lipid accumulation (52% to 19%), suppressed RNF8, restored RXR, and lowered IL-6 and TNF-α levels, indicating strong anti-inflammatory and lipid-modulating effects. Loss of exosomal let-7c-3p aggravates lipid dysregulation and inflammation in COPD by impairing PPAR/RXR signaling. Restoring let-7 expression reverses these effects, highlighting its potential as a diagnostic biomarker and therapeutic target.

## 1. Introduction

Chronic obstructive pulmonary disease (COPD) is a leading cause of global morbidity and mortality, affecting approximately 384 million people and projected to reach 592 million cases by 2050, representing a 23.3% increase from 2020 [1]. It is characterized by persistent airflow limitation due to chronic inflammation and structural damage to the airways and alveoli, manifesting as emphysema and chronic bronchitis [2]. Cigarette smoke, the primary risk factor, induces oxidative stress, generating reactive oxygen species (ROS) that disrupt lipid homeostasis and trigger inflammatory cascades [3,4]. Foamy macrophages, lipid-laden immune cells prevalent in the lungs of COPD patients, particularly smokers, exacerbate disease progression by releasing pro-inflammatory cytokines (e.g., IL-6, TNF-α) and ROS, contributing to alveolar destruction [5,6]. Exosomes, small extracellular vesicles (30–150 nm), mediate intercellular communication by transporting bioactive molecules, including microRNAs (miRNAs), which regulate gene expression post-transcriptionally [7]. Emerging evidence suggests that exosomal miRNAs play a critical role in modulating lipid metabolism and inflammation, offering potential therapeutic avenues for COPD management [8,9]. It is important to note that all experiments and findings in the present study are based entirely on a murine model. While mouse models provide valuable mechanistic insights, their translational relevance to human physiology may be limited, and the results should be interpreted primarily within the context of the animal model.

Extensive research has highlighted the pivotal role of foamy macrophages in COPD pathogenesis. These cells, characterized by excessive lipid accumulation, exhibit impaired immune functions, including reduced antigen presentation, phagocytosis, and cellular chemotaxis, as observed in bronchoalveolar lavage fluid from COPD patients [5,6,10]. Cigarette smoke-induced oxidative stress promotes lipid oxidation, leading to foamy macrophage formation, which disrupts pulmonary lipid homeostasis and amplifies chronic inflammation [11,12]. Exosomes are implicated in COPD progression, with studies showing that exosomes from activated neutrophils or bronchial epithelial cells in COPD patients induce matrix degradation and emphysema-like phenotypes in mice [13,14]. Exosomal miRNAs are key modulators of immune responses and tissue remodeling. For instance, miR-21 in cigarette smoke-exposed bronchial epithelial cell-derived exosomes promotes myofibroblast differentiation, contributing to airway remodeling [14]. The miRNA let-7, frequently downregulated in COPD-associated exosomes, regulates lipid metabolism by targeting the PPAR/RXR pathway, inhibiting Ring Finger Protein 8 (RNF8) to stabilize Retinoid X Receptor (RXR) and enhance lipid catabolism [15,16]. Clinical studies have reported reduced let-7 expression in the sputum and lung tissue of COPD patients, which correlates with disease severity and smoking history [17], as well as in the bronchoalveolar lavage fluid (BALF) of asthma patients [18]. Additionally, let-7 modulates inflammatory pathways by reducing cytokine production in macrophages, suggesting a protective role in COPD [19].

The aims of this study were: (1) to characterize the exosomal miRNA profile of foamy macrophages compared to normal macrophages using miRNA sequencing; (2) to evaluate the functional impact of let-7—selected as a representative miRNA identified in aim 1—on lipid accumulation, RNF8/RXR expression, and inflammatory cytokine production (IL-6, TNF-α) in foamy macrophages through transfection experiments; and (3) to investigate the PPAR/RXR pathway as a key mediator of let-7’s effects in the pathogenesis of COPD. By addressing these objectives, the study aims to provide mechanistic insights into foamy macrophage-driven COPD progression and to establish exosomal let-7 as a potential biomarker and therapeutic target for mitigating lipid dysregulation and inflammation in COPD.

## 2. Results

### 2.1. Foamy Macrophage Formation and Exosome Characterization

**Figure 1:**
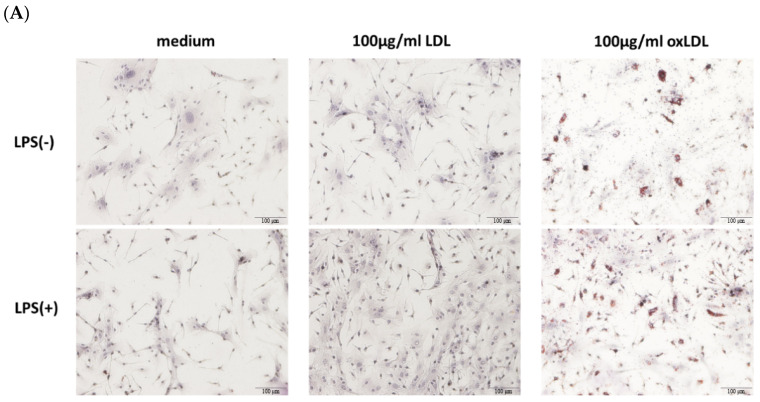

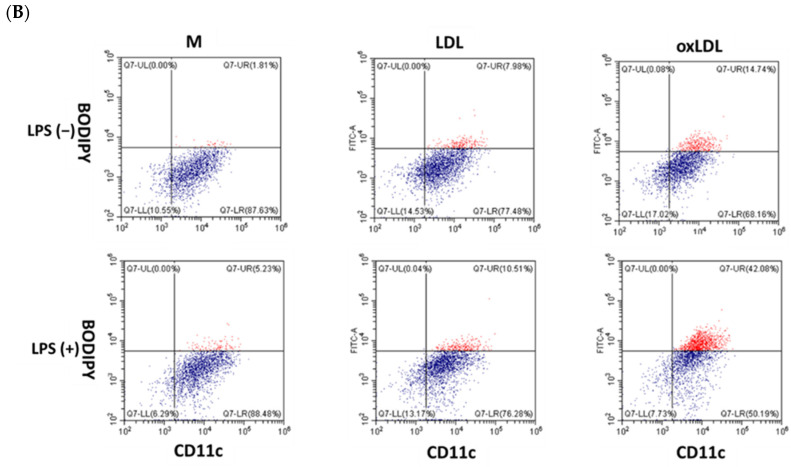
Foamy macrophage formation. (**A**) Oil Red O staining of BMDMs treated with medium (M), LDL (L), oxLDL (O), LPS+medium (LM), LPS+LDL (LL), or LPS+oxLDL (LO). (**B**) Flow cytometry analysis of BODIPY^+^CD11c^+^ (red) foamy macrophages.

Bone marrow-derived macrophages (BMDMs) were treated with medium alone (M), 100 μg/mL low-density lipoprotein (LDL, L), 100 μg/mL oxidized low-density lipoprotein (oxLDL, O), or combinations with 50 ng/mL lipopolysaccharide (LPS): LPS+medium (LM), LPS+LDL (LL), or LPS+oxLDL (LO). Oil Red O staining showed no lipid accumulation in M and L groups, whereas oxLDL treatment (O) induced foamy macrophage formation, with a significant increase in lipid-laden cells in the LO group compared to O (Figure 1A). Flow cytometry using BODIPY and CD11c staining confirmed a higher proportion of foamy macrophages in the O group compared to M and L, with a 27.34% increase in the LO group relative to O, indicating that LPS enhances foamy macrophage formation (Figure 1B).

**Figure 2:**
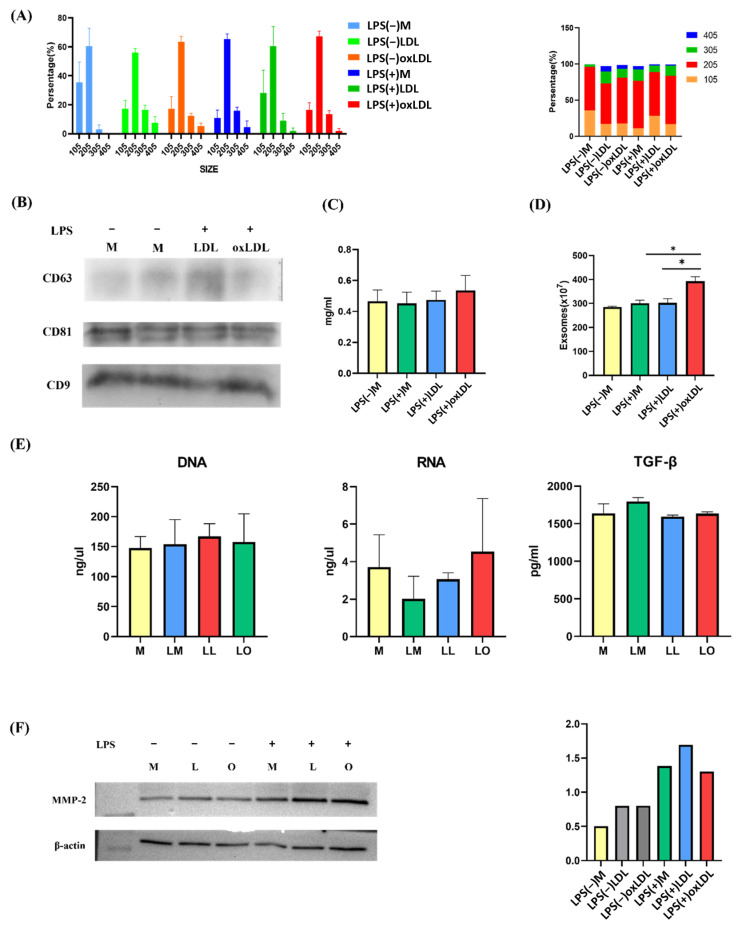
Characterization of exosomes from BMDMs. (**A**) Nanoparticle tracking analysis (NTA) showing EV size distribution. (**B**) Western blotting of exosomal surface markers CD9, CD81, and CD63. (**C**) Protein concentration by Bradford assay. (**D**) Exosome quantification by EXOCET assay. (**E**) DNA and RNA quantification by Nanodrop. TGF-β levels in EVs quantified by ELISA. (**F**) Left panel: MMP-2 expression in exosomes by Western blot. Right Panel: Western blot analysis of MMP-2 expression in exosomes for quantification. Y-axis indicated band intensity. * *p* < 0.05 by Student’s *t*-test.

Exosomes were isolated from BMDM culture supernatants and characterized for size, quantity, and content. Nanoparticle tracking analysis (NTA) revealed similar size distributions across groups, with a slight increase in larger exosomes (>105 nm) in LL and LO groups, but no significant differences between foamy (LO) and non-foamy macrophages (LM, LL) (Figure 2A). Western blotting confirmed the presence of exosome markers CD9, CD81, and CD63 in all groups (Figure 2B). Protein content, measured by the Bradford assay, was slightly elevated in the LO group but not significantly different from M, LM, or LL (Figure 2C). Exosome quantification using the EXOCET assay showed significantly higher exosome numbers in the LO group (392.7 × 10^7^ particles) compared to M (284.9 × 10^7^), LM (301.1 × 10^7^), and LL (302.5 × 10^7^) at equal protein concentrations, confirming increased exosome release by foamy macrophages following LPS and oxLDL stimulation (Figure 2D). Nanodrop analysis indicated no significant differences in total DNA, RNA, or TGF-β content (measured by ELISA) across groups (Figure 2E). The results presented in Figure 2A,C–E were from triplicate biological experiments. Matrix metalloproteinase-2 (MMP-2) expression, assessed by Western blotting (Figure 2F, left panel), was elevated in LPS-treated groups (LM, LL, LO) but similar between LO and LM/LL (Figure 2F, right panel), suggesting no specific association with foamy macrophage formation.

### 2.2. Exosomal miRNA Expression in Foamy Macrophages

**Figure 3:**
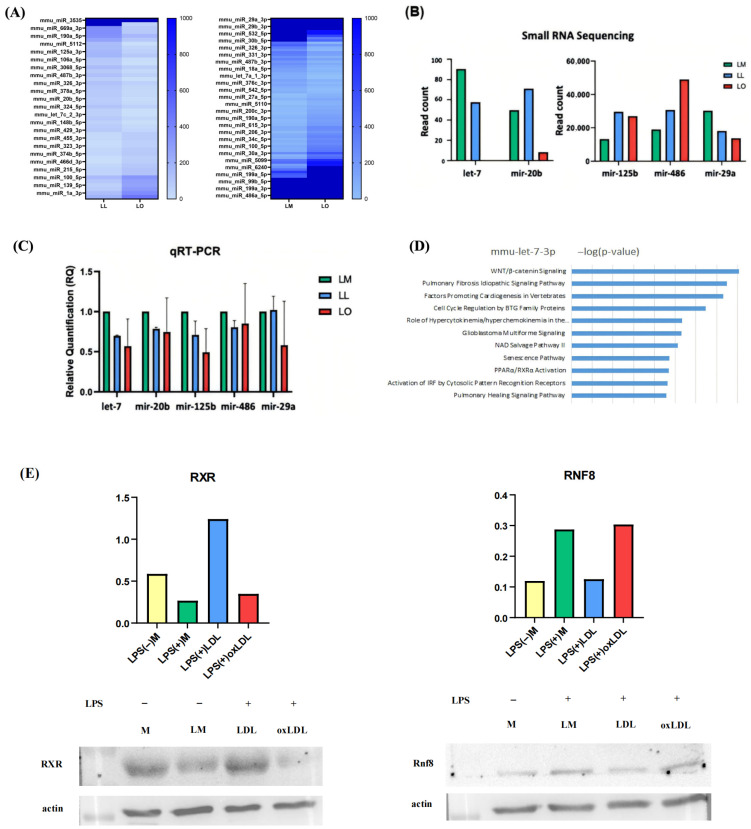
Exosomal miRNA profiling and target prediction. (**A**) Small RNA sequencing analysis identified differentially expressed miRNAs—miR-20b-5p, miR-125b, miR-486-5p, and miR-29a—in exosomes from BMDMs treated with LPS+oxLDL (LO), compared to LPS+LDL (LL) and LPS+medium (LM) groups. (**B**) Read count of identified differentially expressed miRNAs (**C**) qRT-PCR validation confirmed the expression patterns of these miRNAs. (**D**) Pathway enrichment analysis of putative target genes associated with selected miRNAs. Data are presented as bar plots representing enriched biological pathways or signaling cascades. (**E**) Western blot analysis of RXR (left panel) and RNF8 (right panel) protein levels, predicted to be downstream targets of let-7c-3p and associated miRNAs. Upper panels (left and right): Quantification of RXR and RNF8 based on the corresponding Western blots shown below. The Y-axis represents band intensity. Lower panels (left and right): Original Western blot images of RXR and RNF8.

Small RNA sequencing of exosomes from M, LM, LL, LO, and fetal bovine serum (FBS, background control) identified differentially expressed microRNAs (miRNAs). In LO versus LL, 17 miRNAs were upregulated and 29 downregulated (Figure 3A); in LO versus LM, 45 were upregulated and 32 downregulated (fold change > 2, read count difference > 50) (Figure 3B). Five miRNAs (miR-20b-5p, miR-29a-3p, let-7c-3p, miR-125b, miR-486-5p) exhibited significant differential expression. Quantitative real-time PCR (qRT-PCR) validated reduced expression of let-7c-3p and miR-29a-3p in LO exosomes compared to LL, consistent with sequencing results (Figure 3C). Ingenuity Pathway Analysis (IPA) indicated that let-7c-3p targets genes involved in the PPAR/RXR, WNT/β-catenin, and idiopathic pulmonary fibrosis signaling pathways (Figure 3D). Western blotting of BMDMs revealed increased RNF8 and decreased RXR expression in the LO group compared to LL, suggesting impaired lipid metabolism in foamy macrophages (Figure 3E).

### 2.3. Functional Effects of let-7 in Foamy Macrophages

**Figure 4:**
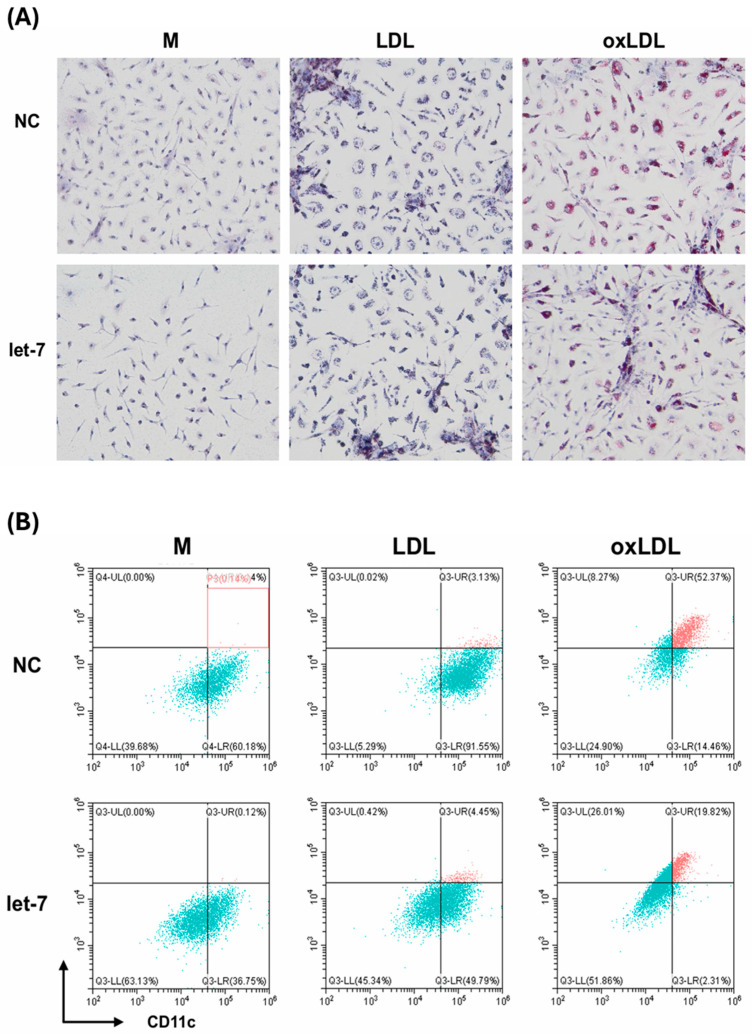
Let-7 reduces lipid accumulation in macrophages. (**A**) Oil Red O staining of BMDMs transfected with let-7 mimics or negative controls and treated with M, LL, or LO. (**B**) Flow cytometry of BODIPY^+^CD11c^+^ cells under same conditions. (red) foamy macrophages.

**Figure 5:**
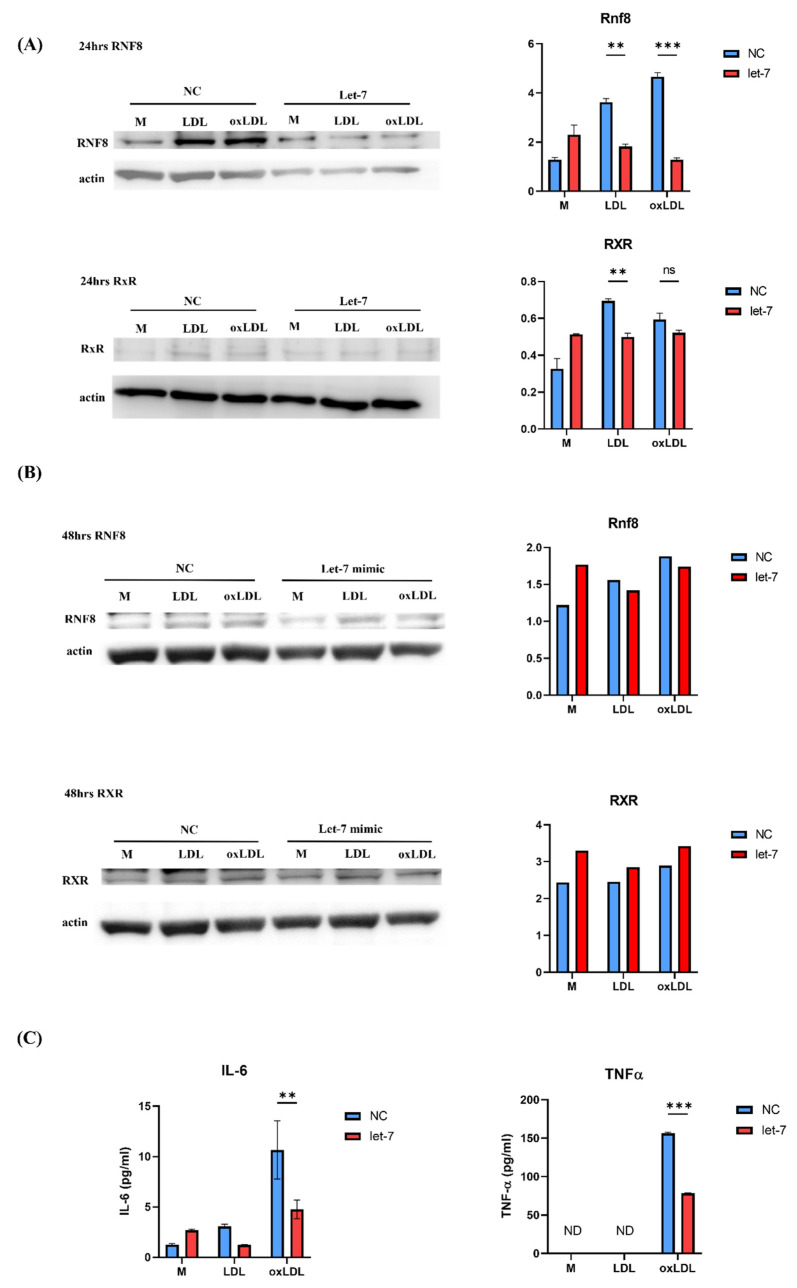
Let-7 modulates lipid metabolism and inflammation. (**A**,**B**) Western blot of RNF8 and RXR in BMDMs transfected with let-7 mimic or control, after 24 and 48 h of LDL or oxLDL stimulation. Y-axis indicated band intensity. (**C**) ELISA quantification of IL-6 and TNF-α in supernatants from BMDMs transfected with let-7 mimics vs. controls. ** *p* < 0.01, *** *p* < 0.001, ns: not significant.

To assess let-7’s functional role, BMDMs were transfected with let-7c-3p mimics or negative control mimics for 48 h, followed by treatment with medium (M), 100 μg/mL LDL (LL), or 100 μg/mL oxLDL (LO) for 24–48 h. Oil Red O staining and flow cytometry showed significant lipid accumulation in the LO group with negative control mimics, which was reduced in the let-7 mimic group (from 52% to 19% foamy macrophages by flow cytometry) (Figure 4A,B). Western blotting at 24 h demonstrated that let-7 mimics suppressed RNF8 expression across all groups and maintained RXR levels in the LO group, in contrast to negative controls where RNF8 increased and RXR decreased (Figure 5A). By 48 h, let-7 mimics further increased RXR expression and reduced RNF8 in the LO group, indicating restored lipid metabolism (Figure 5B). ELISA analysis of culture supernatants showed elevated IL-6 and TNF-α levels in the LO group with negative controls, which were significantly reduced in the let-7 mimic group, suggesting attenuated inflammation (Figure 5C).

## 3. Discussion

Foamy macrophages play a central role in the pathogenesis of chronic obstructive pulmonary disease (COPD), contributing to inflammation and tissue destruction through the release of pro-inflammatory cytokines and matrix metalloproteinases (MMPs) [6,20]. Despite their significance, the mechanisms by which these cells communicate with other cell types—particularly via exosomes—remain insufficiently understood. Exosomes are known to transport miRNAs that modulate immune responses and tissue remodeling. However, the exosomal miRNA profile of foamy macrophages in COPD, including the role of the let-7 family, has not been thoroughly characterized [5,14]. Let-7 is implicated in both lipid metabolism and the regulation of inflammation [9,17], and may influence signaling pathways such as PPAR/RXR. Nonetheless, its role in these pathways within the context of COPD remains largely unexplored, presenting a barrier to the development of targeted therapeutic strategies [11,21].

This study demonstrates that foamy macrophages, induced by oxidized low-density lipoprotein (oxLDL) and further activated by lipopolysaccharide (LPS), release significantly greater quantities of exosomes (392.7 × 10^7^ particles) than non-foamy macrophages (284.9–302.5 × 10^7^ particles). Notably, exosome size and content—including protein, DNA, RNA, TGF-β, and MMP-2—remained unchanged across conditions. Small RNA sequencing revealed distinct miRNA profiles in exosomes from foamy macrophages (LO) compared with those from LPS+LDL-treated (LL) and LPS-treated (LM) macrophages, identifying 17 upregulated and 29 downregulated miRNAs in LO vs. LL, and 45 upregulated and 32 downregulated miRNAs in LO vs. LM. Among these, let-7c-3p emerged as significantly downregulated in LO exosomes. This finding was validated by qRT-PCR and correlated with increased expression of Ring Finger Protein 8 (RNF8) and reduced expression of Retinoid X Receptor (RXR), suggesting impaired lipid metabolism via disruption of the PPAR/RXR pathway. Ingenuity Pathway Analysis (IPA) further supported let-7c-3p’s involvement in regulating PPAR/RXR, WNT/β-catenin, and pulmonary fibrosis signaling pathways.

Functional assays confirmed let-7’s regulatory role: transfection of let-7 mimics into foamy macrophages markedly decreased lipid accumulation (from 52% to 19% lipid-laden cells, measured by flow cytometry), suppressed RNF8 expression, restored RXR levels, and reduced secretion of pro-inflammatory cytokines IL-6 and TNF-α. These results highlight let-7’s dual function in correcting lipid dysregulation and attenuating inflammation.

Although advances have been made in understanding foamy macrophages and exosomes in COPD, significant knowledge gaps persist regarding the specific contributions of exosomal miRNAs—particularly let-7—in modulating macrophage function. While previous studies have reported altered miRNA profiles in COPD, few have focused on the exosomal miRNA content of foamy macrophages or explored their downstream effects on lipid metabolism and inflammatory signaling [14,15,16,17]. The molecular mechanisms by which let-7 regulates RNF8 and RXR expression—and the subsequent impact on PPAR/RXR-mediated lipid catabolism and cytokine production—remain poorly defined. Moreover, the translational potential of let-7 as a diagnostic biomarker or therapeutic target for COPD is under-investigated, with limited studies bridging in vitro findings to clinical relevance [18,19,22].

Our findings indicate that foamy macrophages contribute to COPD progression not only through excessive exosome release, but also by disseminating pro-inflammatory and lipid-disrupting signals within the pulmonary microenvironment. The marked downregulation of let-7c-3p in exosomes from foamy macrophages is consistent with clinical observations of reduced let-7 expression in sputum and lung tissue from COPD patients, especially among smokers, where its levels correlate with disease severity [9,11,17,18,21]. Mechanistically, this reduction in let-7 appears to disrupt lipid homeostasis by enhancing RNF8-mediated RXR degradation, thereby impairing PPAR/RXR-driven lipid catabolism and promoting foamy macrophage accumulation [12].

The increase in RNF8 and concomitant decrease in RXR observed in foamy macrophages underscores a let-7–dependent molecular pathway that exacerbates lipid dysregulation—a key pathological feature of COPD [16]. Importantly, let-7 restoration via mimics reversed these changes: Oil Red O staining and flow cytometry confirmed a 63% reduction in lipid-laden macrophages, RXR expression was restored, and PPAR/RXR signaling enhanced. In addition, IL-6 and TNF-α levels decreased by approximately 40–50% (measured by ELISA), likely through PPAR-mediated suppression of NF-κB activity. These findings support let-7’s therapeutic potential in mitigating both lipid accumulation and inflammation in COPD [11,12,15,16,18,21]. Altogether, our results identify let-7 dysregulation as a key driver of COPD pathology—linking aberrant lipid metabolism with chronic inflammation—and align with broader studies on miRNA perturbation in pulmonary disease [23,24].

Foamy macrophages contribute significantly to the chronic inflammatory milieu of chronic obstructive pulmonary disease (COPD) by releasing pro-inflammatory cytokines such as IL-6, TNF-α, IL-1α, and IL-1β. These cytokines recruit neutrophils and T cells, and activate epithelial cells and fibroblasts, thereby driving airway remodeling, mucus hypersecretion, and emphysema development [25]. Additionally, foamy macrophages generate reactive oxygen species (ROS), which exacerbate oxidative stress and promote alveolar injury [13,26]. Their lipid-laden phenotype, primarily driven by cigarette smoke-induced lipid oxidation, impairs phagocytosis and antigen presentation, further perpetuating inflammation [10,27]. In COPD patients, particularly smokers, foamy macrophages are enriched in bronchoalveolar lavage fluid, with their abundance correlating with disease severity and frequency of exacerbations [5].

Exosomes released from foamy macrophages further amplify inflammation by delivering microRNAs such as miR-223 and miR-21, which respectively promote pro-inflammatory macrophage polarization and fibrotic responses [14,27,28]. In this study, exosome release was 37% higher in foamy macrophages (LO) compared to controls, indicating a potent mechanism for propagating inflammatory signals within the lung microenvironment [13]. Notably, the observed downregulation of let-7 in LO-derived exosomes enhances pro-inflammatory gene expression by lifting repression on NF-κB signaling and cytokines like IL-6 [15]. Restoration of let-7 attenuated the expression of IL-6 and TNF-α, likely through stabilization of RXR and enhanced PPAR-mediated suppression of NF-κB activity, highlighting let-7’s critical anti-inflammatory function in COPD pathogenesis.

The observed downregulation of exosomal let-7c-3p highlights its potential as a diagnostic biomarker for COPD. Quantifying microRNAs such as let-7 in bronchoalveolar lavage fluid or serum-derived exosomes may facilitate early disease detection, assessment of severity, and monitoring of therapeutic response, given its established association with COPD progression [9,17]. From a therapeutic perspective, exosome-based delivery of let-7 mimics could restore lipid metabolism and attenuate inflammation, taking advantage of the natural lung-targeting properties of exosomes [22,29]. Preclinical studies have already demonstrated the efficacy of exosomal miRNA delivery in reducing pulmonary inflammation, supporting the feasibility of this approach [22,29,30].

In addition, pharmacologic activation of the PPAR/RXR axis using agonists such as fenofibrate may complement let-7-based therapies, as PPAR activation has been shown to reduce macrophage-driven inflammation and lipid accumulation [16,21,31]. Let-7’s interaction with surfactant protein D, a known inhibitor of foamy macrophage formation, also warrants further investigation as a potential strategy to restore alveolar homeostasis [5,32].

Building on these results, our findings further suggest that exosomal let-7c-3p regulates inflammatory signaling in COPD. Its downregulation in foamy macrophage exosomes may enhance NF-κB activity through impaired PPAR/RXR signaling, leading to increased IL-6 and TNF-α production and promoting chronic inflammation and airway remodeling [6,15,17]. Restoration of let-7c-3p via mimics reduced cytokine release and lipid burden, supporting its dual anti-inflammatory and lipid-regulatory functions, consistent with reduced let-7 expression in COPD patient samples [17,18]. However, the effects of let-7 are context-dependent. For example, in asthma, let-7 may exacerbate Th2-driven inflammation by targeting IL-10, whereas in pulmonary fibrosis, let-7d inhibits epithelial-to-mesenchymal transition and may act protectively [13,16,24]. These observations underscore the need for cell- and disease-specific strategies when considering let-7-based therapies.

IL-6, markedly reduced after let-7c-3p transfection, is a key cytokine in COPD, driving neutrophil recruitment, STAT3 activation, and airway remodeling via trans-signaling [5,10,27]. Although IL-6 can exert beneficial effects in acute injury or repair contexts [33], in the chronic inflammatory environment of COPD its pathogenic role predominates. Thus, modulation of IL-6 by let-7c-3p highlights a promising therapeutic avenue, but one that requires careful targeting to preserve IL-6’s protective functions in other settings.

From a translational perspective, exosomal let-7c-3p holds potential both as a biomarker and as a therapeutic target. Reduced levels in BALF or serum-derived exosomes may support early diagnosis or severity assessment [17,18]. Exosome-based delivery of let-7 mimics could restore lipid metabolism and suppress inflammation in recipient cells, taking advantage of exosomes’ natural lung-targeting properties [23,27]. In addition, combining let-7 approaches with anti-IL-6 therapies (e.g., sgp130Fc) or PPAR/RXR agonists may further suppress macrophage-driven inflammation while maintaining epithelial repair. Strategies that modulate exosome release or integrate let-7 with established biomarkers such as sputum eosinophils could further enable precision medicine approaches in COPD.

Targeting exosome biogenesis—such as through the inhibition of neutral sphingomyelinase 2—could further mitigate the spread of pro-inflammatory signals by limiting exosomal release [34]. Given its capacity to suppress IL-6, TNF-α, and other inflammatory cytokines, let-7 also holds promise as a therapeutic agent to reduce COPD exacerbation frequency, particularly when used in conjunction with current anti-inflammatory treatments such as corticosteroids or phosphodiesterase-4 inhibitors [35,36].

Integrating let-7 expression profiling with established biomarkers—such as sputum eosinophil counts—could pave the way for precision medicine approaches in COPD, allowing for individualized treatment strategies [37]. Ultimately, let-7’s dual role in regulating both lipid metabolism and inflammation address a fundamental aspect of COPD pathophysiology, making it a compelling and versatile therapeutic target [4].

This study’s strengths include robust exosomal miRNA profiling using small RNA sequencing, qRT-PCR, and let-7 mimic assays, with the BMDM model accurately mimicking COPD conditions [6]. The PPAR/RXR focus provides novel mechanistic insights, supported by consistent results across assays. Limitations include the in vitro model’s limited generalizability to human alveolar macrophages, necessitating in vivo validation [10,13,26]. The focus on let-7c-3p may overlook other miRNAs, and the Exoquick-TC method may introduce impurities [27,38]. Exosome uptake by other lung cells was not explored, limiting intercellular effect insights [13].

## 4. Materials and Methods

### 4.1. Experimental Animals

Female C57BL/6J mice (5–6 weeks old) were sourced from the National Laboratory Animal Center (Taiwan) and maintained at the Experimental Animal Center, China Medical University, under standard conditions with ad libitum access to chow and water. All procedures were approved by the Institutional Animal Care and Use Committee (IACUC No. CMUIACUC-2022-403).

### 4.2. Foamy Macrophage Preparation

Bone marrow-derived macrophages (BMDMs) were isolated from the femurs and tibiae of C57BL/6J mice. Bone marrow cells (5 × 10^6^) were seeded in 10 cm dishes in RPMI-1640 medium supplemented with 10% fetal bovine serum (FBS), 100 U/mL penicillin, 100 μg/mL streptomycin, 1% sodium pyruvate, 25 mM HEPES, 2 mM L-glutamine, and 50 μM 2-mercaptoethanol (all HyClone, Seattle, WA, USA). Differentiation was induced with 10 ng/mL macrophage colony-stimulating factor (M-CSF, PeproTech, Cranbury, NJ, USA) added on days 3 and 6. On day 7, adherent BMDMs were harvested using 0.05% trypsin-EDTA (HyClone), replated at 5 × 10^6^ cells/dish, and rested overnight. On day 8, cells were stimulated with 50 ng/mL lipopolysaccharide (LPS, Sigma-Aldrich, St. Louis, MO, USA) for 24 h. On day 9, cells were treated with 100 μg/mL oxidized low-density lipoprotein (oxLDL, Prospec Tany, TechnoGene, Ltd., Ness-Ziona, Israel), 100 μg/mL low-density lipoprotein (LDL, Prospec), or medium alone for 24 h. Cells were washed twice with phosphate-buffered saline (PBS) to remove residual stimulants, and fresh medium was added on day 10. Culture supernatants were collected after 72 h for exosome isolation.

### 4.3. Oxidized Low-Density Lipoprotein (oxLDL) Preparation

Human LDL (1 mg/mL, Prospec Tany, TechnoGene, Ltd.) was oxidized with 100 μM CuSO_4_ (Sigma-Aldrich) at 37 °C for 16–18 h. The reaction was stopped with 1 mM EDTA (J.T. Baker, Radnor, PA, USA), and the oxLDL was dialyzed against PBS (pH 7.4) using a CelluSep membrane (Membrane Filtration Products, Austin, TX, USA) with four exchanges over 4 h. The solution was filtered through a 0.22 μm PVDF membrane (Merck Millipore, Germany), and protein concentration was quantified using the Bradford Protein Assay (Bio-Rad, Hercules, CA, USA).

### 4.4. Exosome Isolation and Quantification

Exosomes were isolated from BMDM culture supernatants using Exoquick-TC (System Biosciences, Palo Alto, CA, USA) according to the manufacturer’s instructions. Supernatants were centrifuged at 1500× *g* for 15 min at 4 °C to remove debris, mixed with Exoquick-TC reagent (1:5 ratio), and incubated overnight at 4 °C. Exosomes were pelleted by centrifugation, resuspended in PBS, and stored at −80 °C. Exosome quantity was determined using the EXOCET Exosome Quantitation Kit (System Biosciences) with absorbance measured at 405 nm on a SpectraMax iD3 microplate reader (Molecular Devices, San Jose, CA, USA). Protein content was quantified using the Bradford Protein Assay (Bio-Rad).

### 4.5. Nanoparticle Tracking Analysis (NTA)

Exosome size and concentration were assessed using a NanoSight LM10HS (Particle Metrix, Germany) at the Micro and Nanotechnology Research Center, National Cheng Kung University. Exosomes were diluted in PBS to a concentration of 100–200 particles/mL. Polystyrene beads (102 nm, Microtrac, Montgomeryville, PA, USA) were used as a calibration standard before each measurement.

### 4.6. Western Blotting

Proteins were extracted from cells or exosomes using RIPA buffer supplemented with protease inhibitors (Roche, Switzerland). Protein samples (30 μg) were separated on 10–12% SDS-PAGE gels, transferred to PVDF membranes (Bio-Rad), and blocked with 5% non-fat milk in Tris-buffered saline with 0.1% Tween-20 (TBST) for 1 h at room temperature. Membranes were incubated overnight at 4 °C with primary antibodies: anti-CD81 (1:200), anti-CD9 (1:1000), anti-CD63 (1:1000), anti-RNF8 (1:5000), anti-RXRα (1:2500), anti-MMP2 (1:1000), and anti-β-actin (1:1000) (all GeneTex, Irvine, CA, USA). After washing, membranes were incubated with secondary antibodies (goat anti-rabbit or anti-mouse IgG, 1:10,000, GeneTex) for 1 h at room temperature. Protein bands were visualized using Clarity Western ECL Substrate (Bio-Rad) and imaged with a ChemiDoc Imaging System (Bio-Rad). Band intensity was quantified using ImageJ 1.49 software (NIH, Bethesda, MD, USA).

### 4.7. Flow Cytometry

BMDMs (5 × 10^5^ cells/tube) were washed with FACS buffer (PBS with 2% FBS), stained with anti-CD45 and anti-CD11c antibodies (0.15 μg/tube, BD Biosciences, Franklin Lakes, NJ, USA) at 4 °C for 30 min, and then stained with BODIPY (0.38 nM, Thermo Fisher Scientific, Waltham, MA, USA) at 37 °C for 10 min to detect lipid accumulation. Cells were fixed in 2% formaldehyde and analyzed using a CytoFLEX flow cytometer (Beckman Coulter, Brea, CA, USA). Data were processed using FlowJo 7.6 software (BD Biosciences).

### 4.8. Oil Red O Staining

BMDMs were fixed with 10% formaldehyde for 10 min, washed with PBS, and stained with Oil Red O (Sigma-Aldrich) to visualize lipid droplets. The Oil Red O working solution was prepared by diluting stock solution (0.5% in isopropanol) with distilled water (3:2), incubating for 10 min, and filtering through a 0.22 μm membrane. Cells were counterstained with hematoxylin and imaged using an Olympus CKX53 inverted microscope (Olympus, Tokyo, Japan).

### 4.9. miRNA Sequencing

Exosomal RNA was extracted from four groups—unstimulated (M), LPS-stimulated (LM), LPS+LDL (LL), and LPS+oxLDL (LO)—and FBS (background control) using TRIzol Reagent (Ambion, Austin, TX, USA). miRNA libraries were constructed using the QIAseq miRNA Library Kit (QIAGEN, Germany) and sequenced on an Illumina platform generated by Welgene Biotech Co (Taiwan). Raw fastq data were processed with miRDeep2 to remove adapters and low-quality reads, aligned to miRBase21, and normalized using DESeq2. Differentially expressed miRNAs (fold change > 2, read count difference > 50) were identified and analyzed with Ingenuity Pathway Analysis (IPA, QIAGEN) to determine target genes and pathways.

### 4.10. Quantitative Real-Time PCR (qRT-PCR)

Exosomal RNA was extracted using the Total Exosome RNA and Protein Isolation Kit (Thermo Fisher Scientific) and quantified with a Nanodrop ONE spectrophotometer (Thermo Fisher Scientific). RNA (10 ng) was reverse-transcribed into cDNA using TaqMan Small RNA Assays (Thermo Fisher Scientific). miRNA expression was analyzed using a StepOnePlus Real-Time PCR System (Thermo Fisher Scientific) with TaqMan probes for let-7c-3p and miR-29a-3p, normalized to U6 snRNA.

### 4.11. miRNA Transfection

BMDMs (7 × 10^5^ cells/well) were seeded in 6-well plates and transfected with 25 pM let-7c-3p mimics or negative control mimics (Thermo Fisher Scientific) using Lipofectamine RNAiMAX Reagent (Thermo Fisher Scientific) according to the manufacturer’s protocol. After 48 h, cells were treated with medium, 100 μg/mL LDL, or 100 μg/mL oxLDL for 24–48 h. Cells and culture supernatants were collected for downstream analyses.

### 4.12. Enzyme-Linked Immunosorbent Assay (ELISA)

Cytokine levels (IL-6, TNF-α, TGF-β) in culture supernatants or exosomes were quantified using DuoSet ELISA kits (R&D Systems, Minneapolis, MN, USA). A 96-well plate was coated with capture antibody (diluted in PBS according to the manufacturer’s instructions; 100 μL/well) and incubated overnight at room temperature with gentle shaking. Wells were washed three times with PBST (PBS containing 0.05% Tween 20) and blocked with 300 μL/well reagent diluent (PBS containing 1% BSA) for 1 h at room temperature. After washing, 100 μL of either sample or serially diluted standard was added to each well and incubated for 2 h with shaking. The plate was washed, and 100 μL/well of detection antibody (diluted in reagent diluent) was added and incubated for 2 h at room temperature. Wells were then washed and incubated with 100 μL/well Streptavidin–HRP for 20 min in the dark. After washing, 100 μL/well TMB substrate was added and allowed to develop until the lowest standard concentration produced a visible signal. The reaction was stopped with 50 μL/well 2N H2SO4, and absorbance was measured at 540 nm using a SpectraMax iD3 microplate reader (Molecular Devices).

### 4.13. Statistical Analysis

Data were analyzed using GraphPad Prism 8 (GraphPad Software, San Diego, CA, USA). Comparisons between two groups were performed using Student’s unpaired *t*-test. Multiple group comparisons were conducted using one-way ANOVA with Tukey’s post hoc test. For methacholine challenge responses, two-way ANOVA was used. Results are expressed as mean ± standard error of the mean (SEM), with a *p*-value < 0.05 considered statistically significant.

## 5. Conclusions

Exosomal let-7c-3p is a critical regulator of lipid metabolism and inflammation in foamy macrophages, with its downregulation driving COPD progression via impaired PPAR/RXR signaling and enhanced cytokine production. Foamy macrophages amplify inflammation through increased exosome release, propagating pathogenic signals. Let-7 restoration mitigates these effects, offering diagnostic and therapeutic potential. Studying exosomal miRNAs like let-7 could transform COPD management, with future in vivo and clinical studies needed to validate these findings.

## Data Availability

The data that support the findings of this study are available from the corresponding author upon reasonable request.
